# Do they cope or mope? A survey of GPs’ experiences with the changes in the Norwegian Cervical Cancer Screening Programme

**DOI:** 10.1080/02813432.2022.2139481

**Published:** 2022-10-31

**Authors:** Sofie Afseth, Anna Bowe, Bente Prytz Mjølstad, Gunnhild Åberge Vie, Ingrid Baasland

**Affiliations:** a Department of Public Health and Nursing, General Practice Research Unit, Norwegian University of Science and Technology (NTNU), Trondheim, Norway; b Cancer Registry of Norway, Oslo, Norway

**Keywords:** Cervical cancer screening, HPV-based screening, cytology-based screening, general practitioner, HPV

## Abstract

**Objective:**

To explore Norwegian general practitioners’ (GPs) experiences with the changes in the cervical cancer screening programme and to uncover which aspects of the programme they find most challenging.

**Design:**

We conducted an electronic cross-sectional survey.

**Setting:**

Norwegian GPs were invited to participate in the survey between February and September in 2020.

**Subjects:**

One hundred and fifty-five of 429 invited Norwegian GPs responded.

**Main outcome measures:**

Self-reported measures were used to analyse GPs experiences and beliefs related to the screening programme.

**Results:**

Most GPs did not find it particularly challenging to keep up with the changes in the screening programme, regardless of whether they came from areas with HPV-based or cytology-based cervical cancer screening implemented. Challenges concerning the follow-up of patients after an abnormal test were a frequently reported issue. We did not find any differences in how often GPs were uncertain of the follow-up of an abnormal test result in areas with HPV-based compared to cytology-based screening.

**Conclusions:**

The implementation of HPV-based cervical cancer screening in women 34–69 years does not seem to have affected how challenging the GPs perceive the screening programme.

**Key Points**
- How Norwegian general practitioners (GPs) keep up with changes in the Norwegian Cervical Cancer Screening Programme (NCCSP) has not been assessed previously.
- Most GPs did not find it particularly challenging to keep up with changes in the NCCSP regardless of whether they belonged to an area of HPV-based or cytology-based screening.
- The follow-up of patients with an abnormal test result was one of the main challenges reported by the GPs.

## Introduction

Cervical cancer is the fourth most common cancer and cancer-related death in women worldwide [1]. To reduce cervical cancer incidence and mortality, the national Norwegian Cervical Cancer Screening Programme (NCCSP) invites all Norwegian women aged 25–69 to regular screening. Since the programme’s introduction in 1995, the NCCSP has undergone several changes. In 2015, a gradual implementation of HPV testing, replacing cytology as the primary screening method, started for women aged 34–69 years. The implementation has affected different Norwegian counties at different times and was completed in January 2022. The HPV test has a greater sensitivity in detecting precancerous and cancerous lesions in the cervix compared with cytology [2,3]. With the transition to HPV-based screening, the routine screening interval has increased from 3 to 5 years. Young women are frequently infected with HPV [4]; hence, women aged 25–33 years are still offered cytology-based screening every third year [5]. The flowchart for follow-up of abnormal test results was last updated in 2018 [6]. It is more complex as different HPV types have different recommendations regarding follow-up compared to previous charts.

Most Norwegian women get their general practitioner (GP) to perform the screening test. Since the General Practitioner Scheme (a Norwegian patient list system) was introduced in 2001, their workload has increased [7]. When counting guidelines provided by The Norwegian Directorate of Health, it is evident that Norwegian GPs must adhere to almost 80 different national guidelines [8]. Knowing which information is valid at any given time may be challenging. Attempts to adhere to all clinical guidelines can make GPs loose overview, which in turn may result in a tendency to give up on guidelines altogether [9]. A recent Norwegian survey found that three-quarters of GPs felt that they in varying degrees lack sufficient opportunities to stay professionally updated due to their workload [10]. The ongoing changes in the NCCSP require that GPs relate to updated screening guidelines and a more complex flowchart. This study aimed to explore how challenging GPs perceive the changes in the NCCSP and uncover which aspects of the screening programme GPs perceive as demanding.

## Materials and methods

### Study sample

Between February and September 2020, we conducted an electronic cross-sectional survey among 429 Norwegian GPs registered to attend Oppdalsuka, an annual event that offers a wide range of medical courses for both GPs in specialisation and fully trained GP specialists from different parts of Norway. This setting was chosen as attendance to this event presumably was unrelated to knowledge and attitudes towards cervical screening. Recruiting GPs from this event would presumably give us a higher participation rate than distributing the survey by email, as we could promote the survey personally. All registered participants at Oppdalsuka were invited to the survey by email with two additional reminders.

### Questionnaire

The questionnaire was developed by the research group with input from a professional academic group of GPs. A draft of the questionnaire was sent to a small sample of GPs for pilot-testing before a final revision.

An online survey was administrated by using a tool for surveying, registering and collecting data (Nettskjema). The questionnaire included 33 questions. Five questions were open-ended with free text boxes, while the rest were closed-ended items. All closed-ended items had to be answered to complete the survey. Free text answers were analysed for content and categorised before they were used in frequency analysis. All completed surveys (*n* = 155) were included and used in further analyses.

The questions in the questionnaire, relevant for this study, included questions about demographics, GPs clinical experiences with performing cervical sampling, use of recourses regarding the NCCSP and cooperation with gynaecologists. It also contained a section about experiences with implementation of HPV-based screening. These questions were available to GPs from relevant counties only.

### Statistical analysis

Data were analysed in IBM^®^ SPSS^®^ Statistics Version 26 (Armonk, NY). The distribution of answers is presented in numbers and percentages. We used the Chi-square test to determine the statistical significance of differences between different categories. We mainly stratified participants according to demographic characteristics (gender, specialist/in specialisation, years in practice, geographical affiliation). We condensed the geographical affiliation into three groups of counties (HPV-based screening from 2015, from 2019 and no HPV-based screening). We grouped the answers ‘rarely’ and ‘never’ together for question 29 (Appendix 1). Similarly, we grouped the answers ‘to a very small extent’ and ‘to a small extent’, and the answers ‘to a large extent’ and ‘to a very large extent’ in question 17. The level of significance was set at *p* < 0.05.

### Ethics

The study was submitted to the Health Research Committee for Medical and Health Ethics (REK Central Norway), but approval was not considered necessary. It was approved by Norwegian Centre for Research Data (NSD) in February 2020 (reference 463405). Participation in the study was voluntary and anonymous. The GPs consented their participation by answering the questionnaire.

## Results

### Demographics and cervical sampling in clinical practice

Of the 429 invited GPs, 155 (36%) responded to the survey. The sociodemographic characteristics of the study population compared with the total population of GPs in Norway are demonstrated in Table 1. Our sample was notably younger with fewer fully trained specialists and more female GPs compared to the distribution of all GPs in Norway. It also included a greater proportion of GPs already being introduced to HPV-based screening compared to the national distribution.

**Table 1.** Demographics of the GPs in the study (*n* = 155) compared to Norwegian GPs in general (*n* = 4858).

	Respondents, *n* (%)	GPs in Norway^a^, *n* (%)
*Gender*		
Women	92 (59%)	2158 (44%)
Men	63 (41%)	2700 (56%)
*Age*		
<30 years	18 (12%)	97 (2%)
30–39 years	78 (50%)	1334 (28%)
40–54 years	45 (29%)	1970 (41%)
55–66 years	7 (10%)	1247 (26%)
>67 years	3 (4%)	210 (4%)
*Specialists*	47 (30%)	3118 (63%)^b^
*Years in practice*		
<5 years	77 (50%)	–
>5 years	78 (50%)	–
*HPV-based screening introduced*		
HPV-based screening from 2015^c^	52 (34%)	1326 (27%)b
HPV-based screening from 2019^d^	48 (31%)	1229 (25%)b
No HPV-based screening	55 (36%)	2303 (48%)b

^a^ Number based on 4858 GPs. Source: [11].

^b^ Source: [12].

^c^ Counties Rogaland, Hordaland and Trøndelag. HPV pilot from 2015, complete introduction in 2018.

^d^ Østfold, Sogn og Fjordane, Møre og Romsdal, Nordland, Troms and Finnmark.

Of 155 GPs, more than half (68%) reported that they performed a cervix sample weekly (Table 2). Only 5% performed the test yearly or never. Most of the GPs (96%, 95% CI 92–99) usually carried out the test themselves. Women performed cervical samples more often than men, as 87% of the female GPs reported weekly samples versus 41% of the male GPs (*χ*^2^(2)=36.46, Phi = 0.485, *p* < 0.001). Two male GPs commented in free text that although they offered to take the sample, several female patients requested a female physician to do it.

**Table 2.** Demographic distributions and results from main questions for the groups compared.

HPV-based screening	HPV-based screening from 2015^a^, *n* (%)	HPV-based screening from 2019, *n* (%)	No HPV-based screening, *n* (%)
*Demographic distribution*			
Gender			
Women	34 (65%)	20 (42%)	39 (71%)
Men	18 (35%)	28 (58%)	16 (29%)
Age			
<30 years	4 (8%)	7 (15%)	7 (13%)
30–39 years	28 (54%)	24 (50%)	26 (47%)
40–54 years	16 (31%)	13 (27%)	16 (29%)
55–66 years	2 (4%)	4 (8%)	4 (7%)
>67 years	2 (4%)	0	2 (4%)
Specialists	17 (33%)	14 (29%)	16 (29%)
Years in practice			
<5 years	26 (50%)	24 (50%)	27 (49%)
>5 years	26 (50%)	24 (50%)	28 (51%)
Sample frequency			
Weekly	34 (65%)	31 (65%)	41 (75%)
Monthly or less frequent	18 (35%)	17 (35%)	14 (26%)
*Results from main questions*			
The extent of how challenging GPs found it to keep up with the changes in the NCCSP			
To a small/very small extent	20 (39%)	23 (48%)	28 (51%)
To some extent	29 (56%)	19 (40%)	21 (38%)
To a large/very large extent	3 (6%)	6 (13%)	6 (11%)
How often the GPs have to look up or ask a colleague to clarify the follow-up			
Rarely/never	17 (33%)	18 (37%)	18 (33%)
Sometimes	21 (40%)	20 (42%)	21 (38%)
Often	14 (27%)	10 (21%)	16 (29%)

^a^ HPV pilot from 2015, complete introduction in 2018.

### Degree of challenge perceived according to demographics and clinical practice

**Figure 1.**
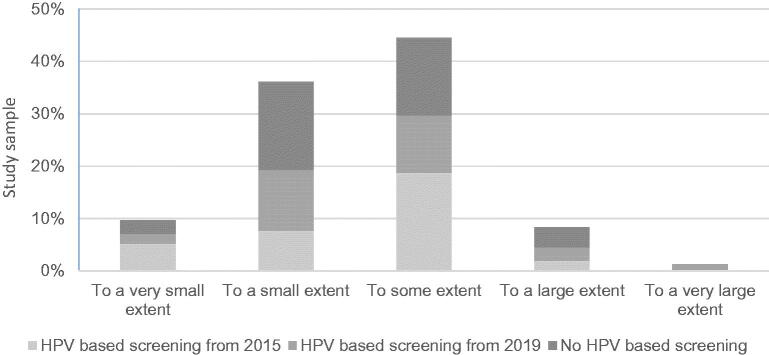
The extent of how challenging GPs found it to keep up with the changes in the NCCSP in percentage of the total study sample.

Only 10% (95% CI 6–16) found it challenging to a large or very large extent to keep up with the changes in the NCCSP. Nearly half of the responders (45%, 95% CI 38–54) found it challenging to a small or very small extent. The level of reported challenges was similar between areas with HPV-based screening and areas with cytology-based screening (*χ*^2^(4)=4.75, *p* = 0.314) (Figure 1).

Performing cervical screening weekly was associated with perceiving the changes substantially less challenging (*χ*^2^(2)=15.5, Cramer’s *V* = 0.316, *p* < 0.001) (Table 3). Fully trained specialists tended to report that they found it challenging to a smaller degree than non-specialists. However, this finding was not statistically significant (*χ*^2^(2)=5.3, Cramer’s *V* = 0.186, *p* = 0.068).

**Table 3.** Degree of challenge to keep up with the changes in the cervical cancer screening programme according to screening sampling frequency and specialist status.

	Degree of challenge to keep up with the changes		
	To a very small/small degree	To some degree	To a very large/large degree
Sample frequency, *n* (%)			
Weekly	58 (55%)	43 (41%)	5 (5%)
Monthly or less frequent	13 (27%)	26 (53%)	10 (20%)
Specialist status, *n* (%)			
Specialist	26 (55%)	20 (43%)	1 (2%)
Non-specialist^a^	45 (42%)	49 (45%)	14 (13%)

^a^ Including participants who reported to be either GPs in specialisation (*n* = 61) or other specialist status (*n* = 7).

### Information-seeking in clinical practice

A quarter of the GPs reported that they often looked up or asked a colleague to clarify the follow up of a cervical sample (26%, 95% CI 19–33), while the majority did it sometimes (40%, 95% CI 27–42) and one-third rarely or never (34%, 95% CI 19–33) (Table 2). There was no significant difference between areas with HPV-based screening compared to areas with cytology-based screening (*χ*^2^(4)=1.02, *p* = 0.907), nor the qualification level (*χ*^2^(3)=5.65, *p* = 0.160).

When facing a challenge related to the NCCSP, the majority (79%, 95% CI 71–85) reported that they use an online medical encyclopaedia made for Norwegian health practitioners, Norwegian electronic medical handbook, while 27% (95% CI 20–34) reported this as their primary source. The Norwegian electronic medical handbook also dominated (95%, 95% CI 90–98) as the preferred website to find updated information about the programme.

Regarding the follow-up of patients after being referred to a gynaecologist for further examination or treatment of abnormal cytology tests, most GPs (86%, 95% CI 79–91) reported that they receive sufficient information from the gynaecologists. Most GPs (82%, 95% CI 75–88) answered that an outlining for the follow-up by the gynaecologist would make them more confident in these cases. Better information from the laboratory followed with 37% (95% CI 30–46), while 14% of the GPs (95% CI 9–21) felt confident enough.

### Information to patients

Among the 100 participants who worked in counties with implemented HPV-based primary screening, 75% (95% CI 65–83) thought that their patients were not informed about the new screening method. Although most GPs (64%, 95% CI 54–73) routinely informed their patients about the changes, one-third (36%) (95% CI 27–47) did not.

### Perceived challenges in relation to the NCCSP

Eighty-eight participants (57%) responded to an open-ended question on what they believed were the largest professional challenges related to the NCCSP today. Concerns regarding the correct medical follow-up of patients after an abnormal test were reported most frequently (*n* = 15). Some GPs from counties with HPV-based screening, specified that the flowchart and guidelines were too complex to understand. Others specified that they found it challenging to deal with the follow-up of those who have had cell changes previously.

Other key themes included difficulties motivating women to participate in the screening programme (*n* = 13) and patients’ expectations of referral to a gynaecologist for routine screening test (*n* = 10). Challenges related to the transition from cytology- to HPV-based screening (*n* = 8) included difficulties explaining why the screening interval had increased from three to five years and difficulties interpreting HPV test results when being used to interpret cytology results. Furthermore, two GPs expressed that it was incomprehensible that cytology had been replaced with HPV testing. Some male GPs (*n* = 6) reported that it was a challenge to maintain cervical screening as a clinical skill. Several respondents also reported in free texts that they did not experience any professional challenge with the programme (*n* = 10).

## Discussion

### Principal findings

In our study, most GPs did not find it particularly challenging to keep up with the changes in the NCCSP, regardless of whether they came from areas with implemented HPV-based screening or from areas with cytology-based screening. Nevertheless, some challenges were identified, and these were mainly similar for GPs from both areas. The follow-up of patients after an abnormal test result was one of the main reported challenges.

### Strength and limitations

This is one of few studies investigating GPs’ experiences with the transition from cytology to HPV-based screening, giving a valuable insight into how GPs respond to the NCCSP changes. Even though health services in other countries vary both in terms of primary health care services and organising of cervical screening, some aspects of this study could be useful for other countries in the transition to HPV-based primary screening.

The results should be interpreted with caution, as the sample size is limited, making it difficult to present any conclusive findings. Compared to the national distribution of GPs, a greater proportion of our sample had been introduced to HPV-based screening, increasing the possibility to identify challenges related to the new screening test and flowchart. However, with a rate of participation of 36%, there risk of selection bias is evident. The participating GPs might be more interested in cervical screening than GPs who chose not to participate. Hence, we cannot know whether those who participated perceived NCCSP and its’ recent changes differently from those who did not. Our study population was younger compared to all Norwegian GPs, which may have affected the results. Our findings also suggest that specialist status may be associated with less perceived challenges to keep up with changes. Also, the study had a greater proportion of female GPs who may have more experience with gynaecological examinations and cervical screening than male GPs, as further discussed below. The generalisability of our results is therefore uncertain. Studies with a higher participation and random sampling of GPs would provide more certainty.

Our findings were also limited by the fact that all data relied on self-reported opinions with no objective confirmation of the data. Also, the open-ended responses we received were short written contributions, more susceptible to misinterpretation.

### Findings in comparison with existing literature

A recent study focusing on the similarly renewed cervical cancer screening programme in Australia, reported that GPs had problems understanding the new guidelines and found the primary screening approach too complex [13]. Similar findings are also presented in another recent Australian study in which more than one-third of screening providers were not comfortable managing positive test results for oncogenic HPV types other than type 16/18, and 15% were not comfortable managing HPV type 16/18 positive test results post-renewal [14]. As in Australia, the Norwegian national flowchart has changed, now including different follow-up for HPV 16/18 compared to other oncogenic HPV types. Even though we did not examine which specific aspects of the HPV-follow-up the GPs found most challenging, our results still reflect a general uncertainty regarding the flowchart. The magnitude of inadequate follow-up of abnormal cervical screening results in Norway is currently unknown, but a recent American study estimated that as much as half of patients with discordant HPV and cytology results do not receive follow-up according to guidelines [15]. In Denmark, guideline-accordant follow-up of cervical screening exit tests was lower among women with previous abnormal test results [16]. Delegating follow-up of women who need individual assessments to gynaecologists with colposcopy expertise who also have access to all test results, could ease correct follow-up and alleviate GPs of their largest reported challenge with the NCCPS. However, follow-up by gynaecologists may not increase guideline adherence [16], and the cost-effectiveness of changing the scheme would have to be evaluated.

Some GPs in our study reported the transition from cytology- to HPV-based screening as the largest challenge. Others found it challenging to explain to patients why the guidelines recommend screening every 5th instead of every 3rd year with HPV testing, suggesting that some GPs still need more information about HPV-based screening. Supporting this is a Norwegian study from 2017, which found that knowledge of the causal relationship between HPV and cervical cancer was relatively low among GPs in Northern Norway [17]. These data were collected prior to the transition to primary HPV screening, yet after the national introduction of the HPV vaccine. The study also revealed that the participating GPs’ self-reported knowledge was higher than their actual knowledge. Our study was based on self-report, and it is possible that the GPs were not aware of their lack of knowledge about HPV and HPV-based screening.

We had expected more GPs to mention the challenge of explaining why the screening interval has increased, as several studies have found that patients’ concerns and limited time to discuss risk and benefits of a longer screening interval, were key reasons why providers did not follow the recommended guidelines for cervical screening [14,18,19].

Although three-quarters of the GPs from areas with HPV-based screening doubted that patients were aware of the new screening method, more than a third did not routinely inform their patients that they were screening for HPV. We did not explore the reasons for not informing. It could be due to time pressure in clinical practice or because they do not see the benefit. A positive HPV result has a psychosexual impact [20], and GPs may be hesitant to provide such information to minimise this. Previous research has shown that women undergoing primary HPV testing may have additional information needs after receiving their results, requesting more information about epidemiology and cause of HPV [21]. Most of the women in an Australian study felt that they had not been provided with enough information about the renewed screening programme [22]. In the same study, the GP was reported as the preferred source for future information on the topic.

Some male GPs in our study expressed that many patients preferred the sample to be taken by a female physician. Other male GPs reported that they do not get the opportunity to perform the test often enough to maintain this clinical skill. In an Australian study, three-quarters of women preferred to see a female practitioner for a Pap smear [22]. A Norwegian study found that male GPs more often omit performing a gynaecological examination [23]. Insecurity regarding cervical screening due to little practice might be a contributing factor to this. A higher threshold for performing a gynaecological examination could also lead to fewer natural situations for men to take the initiative for a screening test, hence less practice in the procedure.

### Interpretation and implications

The introduction of HPV-based screening in Norway has been a gradual process including pilot testing. Piloting is a recommended strategy in the development of successful screening programmes [24] and could explain why the GPs did not find the changes particularly challenging. The screening test is also an established and frequently performed routine test, and our findings showed that the GPs who performed the test more frequently, perceived the changes with greater ease. The test procedure itself has not changed and may reduce the GPs perception of change. Additionally, the laboratories and gynaecologists often recommend a time interval for the follow-up of abnormal test results, streamlining the task for GPs. Our study found that most of the GPs received sufficient information from the gynaecologist. Sometimes, the laboratories might only refer to the guidelines or the clinician’s judgement in more complicated cases with previous abnormal cervical cytology. Some GPs in our study reported these cases as the largest challenge related to the NCCSP. Hence, it appears to be important that the Norwegian laboratories and gynaecologists are consistent in providing an outline of the recommended follow-up with the patients’ test results.

Our study revealed that most GPs use the same information source, the Norwegian electronic medical handbook, to find updated information on the NCCSP. The handbook provides information on most medical topics with integrated national guidelines. According to the handbook own statistics, 95% of Norwegian GPs subscribe to it and 62% use the handbook daily [25]. Based on this report, it seems crucial to ensure that this is always professionally updated.

Finally, it seems evident that women in general and some GPs still need more information about HPV-based screening. This could be a subject for further research. Regardless, it is important to continue the work of informing GPs about HPV-based screening, so they in turn can inform women taking part in cervical cancer screening adequately.

## Appendix 1

### Study questionnaire

Questions from the questionnaire used in this study, excluding questions about demographics (1–9).

10. A woman requests a routine cell sample as part of the NCCSP – what do you usually do?

[ ] Take the sample yourself
[ ] Ask another colleague to take it
[ ] Request a midwife to take it
[ ] Send a referral to a gynaecologist
[ ] Other: Voluntary free text

11. How often do you take a cell sample from the cervix yourself?

[ ] Weekly
[ ] Monthly
[ ] Annually
[ ] Never

17. To what degree do you, as a GP, find it challenging to keep up with the changes in the NCCSP?

[ ] To a very large degree
[ ] To a large degree
[ ] To some degree
[ ] To a small degree
[ ] To a very small degree

18. What sources and strategies do you use when you face professional challenges in connection with the NCCSP?

*Select 1–3 options*

[ ] Norwegian Electronic Medical Handbook (NEL)
[ ] The Cancer Registry/The NCCSP’s own website
[ ] Helsebiblioteket.no
[ ] HelseNorge.no
[ ] The national action program with guidelines for gynaecological cancer (The Norwegian Directorate of Health’s website for guidelines)
[ ] Discuss with a colleague at the GP's office
[ ] Consult with a gynaecologist (dialogue message, telephone, etc.)
[ ] Refer to gynaecologist
[ ] Other: Voluntary free text

19. Which source or strategy do you use the most when you face professional challenges in relation to the NCCSP?

[ ] Norwegian Electronic Medical Handbook (NEL)
[ ] The Cancer Registry/The NCCSP’s own website
[ ] The Health Library’s website (Helsebiblioteket.no)
[ ] HelseNorge.no
[ ] The national action program with guidelines for gynaecological cancer (The Norwegian Directorate of Health’s website for guidelines)
[ ] Discuss with a colleague at the GP's office
[ ] Consult with a gynaecologist (dialogue message, telephone, etc.)
[ ] Refer to gynaecologist
[ ] Other: Voluntary free text

20. Where do you want the information of the changes in NCCSP to be published? More than one answer is possible.

[ ] Norwegian Electronic Medical Handbook (NEL)
[ ] The Cancer Registry/The NCCSP’s own website
[ ] Helsebiblioteket.no
[ ] HelseNorge.no
[ ] The national action program with guidelines for gynaecological cancer (The Norwegian Directorate of Health’s website for guidelines)
[ ] Guideline in gynaecological cancer (Norwegian Gynaecological Association)
[ ] Other: Voluntary free text

21. What do you perceive as the largest professional challenges related to NCCSP today, seen from the GP's office?

[ ] Voluntary free text

29. When you receive an abnormal test result - how often do you have to look up or ask a colleague to clarify the follow-up?

[ ] Often
[ ] Occasionally
[ ] Rarely
[ ] Never

30. When a woman has been to a gynaecologist for an examination or treatment of cell changes and she is going to you for a check-up, do you find that you get sufficient amount of information on the follow-up from the gynaecologist?

[ ] Yes, always
[ ] Normally
[ ] Rarely
[ ] Never

31. What would make you more confident in the correct follow-up of a woman who has had cell changes? *More than one answer is possible.*

[ ] That the gynaecologist outlines the further follow-up in the epicrisis
[ ] Better information from the laboratory
[ ] Not applicable (I feel professionally confident)
[ ] Other: Voluntary free text

32. To what extent do you feel that your patients are informed about the changes in the screening program?*

[ ] To a very large extent
[ ] To a large extent
[ ] To some extent
[ ] To a small degree
[ ] To a very small extent

33. When a patient in the aged 34–69 years requests a cell sample, do you unsolicited inform the patient that they are now being screened for HPV virus, not just cell changes?*

[ ] Yes, for most patients
[ ] For some patients
[ ] No

*Exclusive questions to GPs belonging to counties where HPV testing had been implemented.

Questions from the questionnaire that were not used in the study

12. In which situations have you as a GP taken the initiative to take a cervical sample as part of the screening program in the last year? *More than one answer is possible.*

[ ] Rarely or never the initiative
[ ] Pregnancy checkup and/or postpartum control
[ ] Other gynaecological examination (including IUD insertion)
[ ] During a consultation regarding contraception
[ ] When the woman has had cell changes in the past
[ ] As part of a health check

13. What do you experience as a GP are women’s most common reasons for not taking a cell sample?

*More than one answer is possible.*
[ ] Do not prioritise spending time on the examination
[ ] Reluctance to undergo gynaecological examination
[ ] Reluctance because of male GP
[ ] Due to the woman’s cultural background
[ ] Do not believe in the effect of the screening programmes
[ ] Do not think she is in risk of developing cervical cancer
[ ] Is not familiar with the screening programme
[ ] Other: Voluntary free text

14. What do you as a GP think is the most important measure to improve the participation of NCCSP?

[ ] Voluntary free text

15. Should cervical screening be a part of the GPs tasks?

[ ] Totally agree
[ ] Partly agree
[ ] Neutral
[ ] Partly disagree
[ ] Totally disagree
[ ] Do not know

16. To what extent do you agree in the following statements

**Table:**

	Totally agree	Partly agree	Neutral	Partly disagree	Totally disagree	Dugfzz2so not know
Cervical screening is a natural part of the GPs responsibility	[ ]	[ ]	[ ]	[ ]	[ ]	[ ]
Cervical screening performed by midwives will contribute to an unfortunate development of specialisations in the primary health care	[ ]	[ ]	[ ]	[ ]	[ ]	[ ]
Cervical screening should be the responsibility of primary health care the primary health care as part of the principles of lowest effective level of care	[ ]	[ ]	[ ]	[ ]	[ ]	[ ]
The organisation of NCCSP, which is based on the GPs taking the cervical sample, contributes to GPs maintaining the necessary competence in gynaecological examination	[ ]	[ ]	[ ]	[ ]	[ ]	[ ]
The NCCSP contributes to GPs having a comprehensive knowledge of women’s health in a life course perspective	[ ]	[ ]	[ ]	[ ]	[ ]	[ ]
Cervical screening at the GP provides an opportunity to get into other topics concerning the abdomen and women’s health that would not be possible without the cervical sample	[ ]	[ ]	[ ]	[ ]	[ ]	[ ]

22. Have you taken The Norwegian Medical Associations online course ‘Screening for cervical cancer’?

[ ] Yes, once
[ ] Yes, several times
[ ] No

23. Do you know if the women get a reminder from The Cancer Registry to take a cervical sample?

[ ] Yes
[ ] No

24. Do you inform the patients of the results of the cervical sample?

[ ] Yes, also when the test is normal
[ ] Only with abnormal test results

25. How do you normally inform the patients of an abnormal test result?

[ ] Letter by post
[ ] SMS/patient post
[ ] Call them
[ ] Call in for a consultation

26. Do you use a standard template when you inform the women of the test result?

[ ] Yes, regardless of the answer
[ ] Yes, only when test is normal
[ ] No

27. Do you use The NCCSP’s own website?

[ ] Yes
[ ] No

28. Do you know that the NCCSP has information to both health workers and patients on its website, as well as suggestions for standard templates that can be used for patient information?

[ ] Yes, I use it actively
[ ] Yes, but I rarely use the website
[ ] No, I do not know about the website
